# Establishing an early indicator for data sharing and reuse

**DOI:** 10.1002/leap.1586

**Published:** 2023-11-14

**Authors:** Agata Piękniewska, Laurel L. Haak, Darla Henderson, Katherine McNeill, Anita Bandrowski, Yvette Seger

**Affiliations:** 1 SciCrunch, San Diego, California, USA; 2 Mighty Red Barn, Townsend, Wisconsin, USA; 3 FASEB, Rockville, Maryland, USA; 4 Independent Researcher, Massachusetts, USA

## Abstract

Funders, publishers, scholarly societies, universities, and other stakeholders need to be able to track the impact of programs and policies designed to advance data sharing and reuse. With the launch of the NIH data management and sharing policy in 2023, establishing a pre-policy base-line of sharing and reuse activity is critical for the biological and biomedical community. Toward this goal, we tested the utility of mentions of research resources, databases, and repositories (RDRs) as a proxy measurement of data sharing and reuse. We captured and processed text from Methods sections of open access biological and biomedical research articles published in 2020 and 2021 and made available in PubMed Central. We used natural language processing to identify text strings to measure RDR mentions. In this article, we demonstrate our methodology, provide normalized baseline data sharing and reuse activity in this community, and highlight actions authors and publishers can take to encourage data sharing and reuse practices.

## INTRODUCTION

Over the last 20 years, there has been a growing recognition of the benefits of sharing and reuse of research data: enhancing research transparency, supporting rigour and reproducibility, promoting innovation, and maximizing the economic return on investment of research funding (Vasilevsky et al., 2013: Beagrie & Houghton, 2014; Menke et al., 2022; Starr et al., 2015). Most researchers want to share and reuse data but do not have the time, resources, motivation, or know-how to do so (Hahnel et al., 2020), and rates of data sharing and reuse vary widely among researchers in the biological and biomedical sciences (Park, 2022). The NIH Data Management and Sharing policy (National Institutes of Health, 2020) furthers the requirements for the adoption of data sharing and reuse by the biological and biomedical research community.

Scholarly societies play a vital role in promoting and enabling data sharing and reuse among researchers (Maienschein et al., 2018; Ruediger et al., 2022). The Federation of American Societies for Experimental Biology (FASEB) recently launched DataWorks! (FASEB, 2021), a suite of programs designed to promote, enable, and reward a culture of data sharing and reuse across the biological and biomedical sciences. In 2022, FASEB publications similarly started to require authors to provide data availability statements and require data citation as an initial step on the path to encouraging data sharing and reuse. To assess the impact of these programs, FASEB identified a need to establish a baseline and to monitor changes in data sharing and reuse over time.

A major structural challenge has been how to measure such adoption of data sharing and reuse practice. One option that has been reported separately is to examine data availability statements. During the time period of our study, about 20% of biomedical preprints and published works included such a statement, and very few described openly available data (McGuinness & Sheppard, 2021).

Another option that has also been explored is to examine citation of data in the reference list of research articles (Parsons et al., 2019). Authors may cite data they collected or data they obtained from another source and reused. Data citation standards have been developed and there has been a concerted attempt to align standards and policies (Altman & Borgman, 2015; Cousijn et al., 2019; Data citation principles, 2016; Hrynaszkiewicz et al., 2020). For example, researchers may deposit their data sets into a repository and obtain a unique identifier (DOI) to enable citation and discovery. DataCite Event Data can be used to track citation of those data sets (DataCite, 2022).

However, while data citation infrastructure exists, the adoption of data citation practices is just emerging in the life sciences (Robinson-García et al., 2016). Researchers are starting to deposit their data in repositories, and the implementation of citation practices by publishers is only just emerging (Cousijn et al., 2018). While we would have liked to measure data sharing and reuse using DataCite Event data to track data citations, either directly or through a service such as Scholix (Burton et al., 2017), this approach is not presently feasible (Khan et al., 2020). Illustrating this lag, an August 2022 query using the DataCite Event Data API^1^ showed that there were 5,854 DOIs registered in 2020 with DataCite of type ‘data set’ with at least one citation, the majority of which were registered post-publication by repositories including disciplinary preprint servers and university repositories show-casing faculty works (not by publishers). By comparison, the entire 2020 DataCite Event set had over a million citations, over 95% of which were associated with a single repository. The recent launch of the Open Global Data Citation Corpus by DataCite and partners, which will include DOI and non-DOI data citations will go a long way toward addressing these issues (Vierkant, 2023).

We therefore decided to test an alternative early indicator of data sharing and reuse that could be used to establish baselines and in the time when a more formal citation infrastructure is being adopted. Authors mention research resources, databases, and repositories (RDRs) in the Methods section of journal articles (Park et al., 2016), and there has been some work to track data sharing and reuse practices using a combination of both formal citations and informal references to data within the text of a publication (Park & Wolfram, 2017). RDRs are collated and curated data outputs from many research studies, and include bibliographic databases like Cochrane Library and PsychInfo; reagent databases like ATCC and AddGene; research databases like Ensebl and Pfam; and research software databases and repositories like Cytoscape and MaxQuant. Our hypothesis is that, if we cannot yet measure citation of an individual data set, maybe we can start to understand the potential of data citation infrastructure by measuring RDR citations in their stead.

We describe an approach to measuring biomedical data sharing and reuse that uses tools to mine free text for RDR mentions combined with the SciCrunch database of biological and biomedical research resources used and continually developed by the RRID project (Bandrowski et al., 2015). We present the methodology and descriptive statistics, and discuss the utility and limitations of the approach for assessing the volume of RDR mentions overall as well as more granular measures by resource type, journal, or discipline.

## METHODS

After determining that journal article reference lists are not yet a feasible source for data citations, we decided to focus on text analysis of Methods sections. While authors may list research resources in other sections of a paper, we decided to focus on Methods to reduce the possibility of a false positive if an author were to mention a resource that is not used in the context of the research reported. We obtained Methods text for biological and biomedical journal articles from articles indexed in PubMed and available in the PubMed Central Open Access subset (NLM, 2022) for the years 2020 and 2021. For the purposes of this study, mineable text is dependent on both the licence of the publication as well as whether its journal uses a standard markup language (JATS, the Journal Article Tag Suite) so that sections of the publication are marked and thereby easily queried (see, e.g., Mietchen, 2015). According to EuropePMC, in 2020 there were 1,638,399 articles published and 625,338 (38%) have a Methods section available to text mine (‘free to read and use’).^2^

We identified a discrete subset of SciCrunch RDRs to include in this project. We reviewed the top 1,000 entries in the SciCrunch database, measured by citations, removed entries for organizations (such as universities without a corresponding RDR) or non-relevant tools (such as reference managers), updated links, and consolidated duplicates resulting from RDR mergers and name variations. The resulting list of 737 RDRs is shown in Table S1.

We used harvesting processes to extract RDR mentions based on the RRID initiative methodology (Bandrowski et al., 2015). We also harvested mentions of the URL or name of an RDR listed in the SciCrunch database as described in Ozyurt et al. (2016). This data set was augmented by articles in PubMed Central but not the OA subset in which RRIDs were entered by authors during the journal publication process. To ensure integrity of the harvested data, we performed statistical tests to determine if the RRID citations are consistent with algorithm-found citations. We manually viewed and removed inaccurate outliers, then statistically adjusted the rate of use.

## RESULTS

From the mined Methods text, we extracted RDR mentions and created a unique association between an RDR (represented by an RRID number) and an article where the repository was mentioned (PMID number). For each pair we built a record that consists of:

- RRID of the RDR, name of the RDR (‘record-pair’).
- PubMed Identifier (PMID) of the article, title of the publication, DOI, date of publication, and the snippet (the relevant portion of the author’s sentence describing the repository).
- Title of the journal, journal ID, journal ISSN, and/or journal ESSN.

The resulting 2020 data set consists of 95,430 unique record-pairs; 66,187 unique articles; and 616 unique RDRs; the 2021 data set consists of 110,048 unique record-pairs, 75,532 unique articles, and 619 unique RDRs (Table 1). The data set of all records is available in Table S2.

### RDR mentions

**FIGURE 1:**
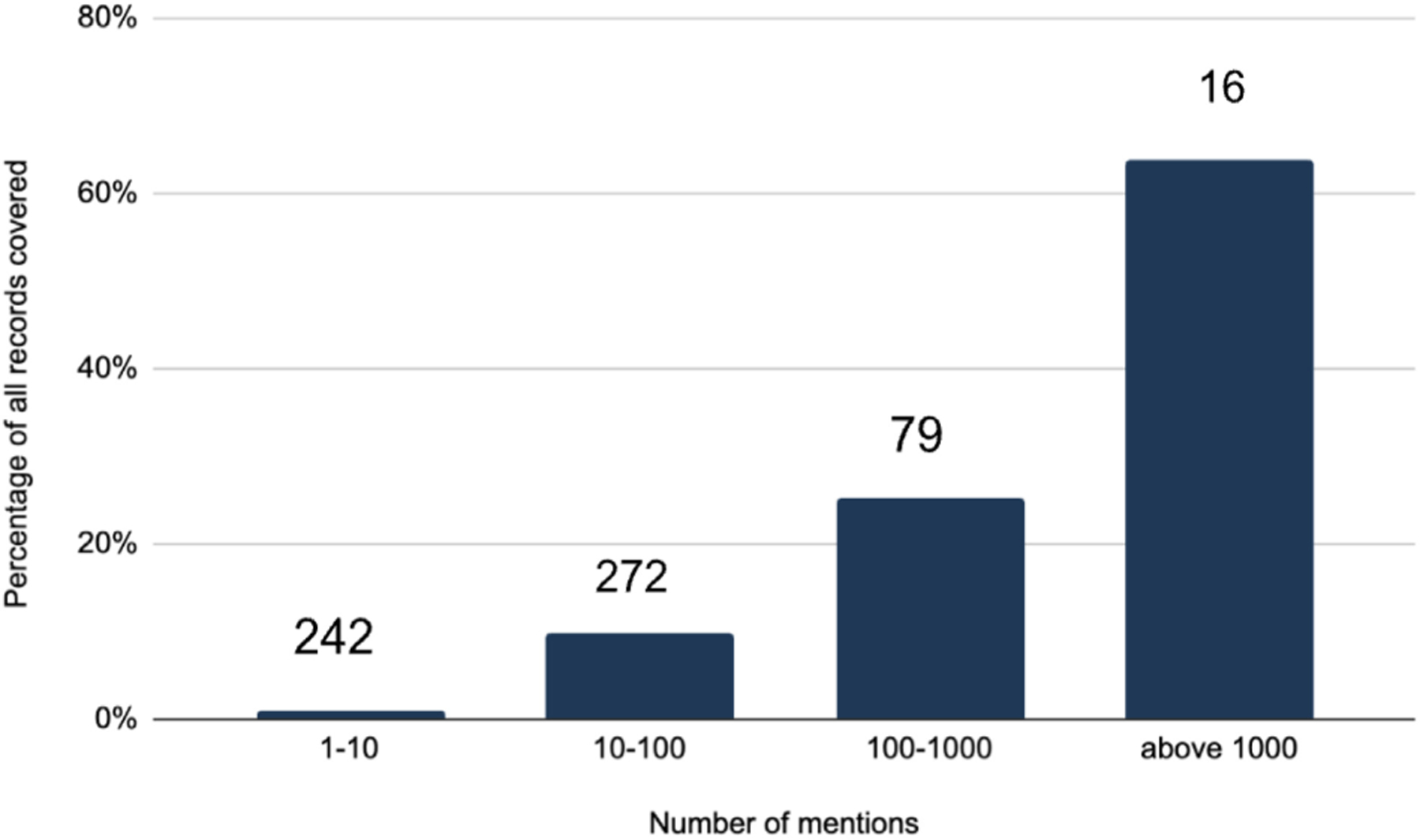
Unique research resources, databases, and repositories (RDR) mentions in 2020, shown as a percent of record-pairs by range of total mentions. The number above each column represents the number of repositories in each range.

We performed a descriptive analysis of the RDRs mentioned to better understand if there are specific journals, research fields, or RDR types that are more frequently mentioned. Overall, the distribution of RDR mentions is a long-tail type of distribution: most articles refer to a relatively small group of RDRs, while most RDRs are mentioned relatively infrequently (Fig. 1).

The most frequently mentioned RDRs are shown in Table 2, together with the discipline, RDR type, number of record-pairs, and list rank for 2020 and 2021. The ten most-mentioned RDRs covered over half of all mentions, and the 20 most-mentioned covered 65% of all mentions.

**FIGURE 2:**
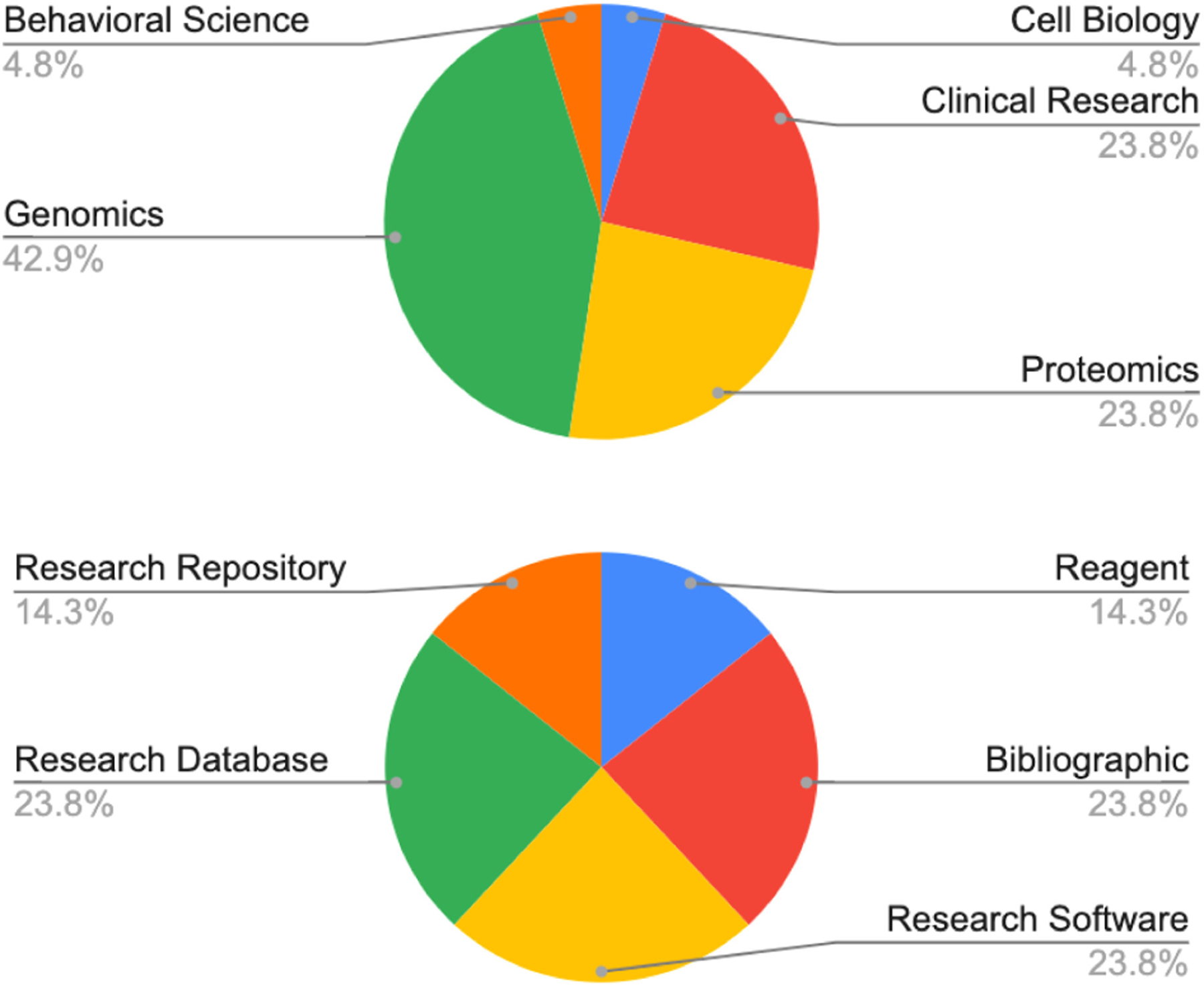
Distribution of research resources, databases, and repositories (RDRs) in the top 20 mentions by research area (*top*) and type (*bottom*). *Research databases*: databases containing aggregated structured research data; for example, databases of ontological (GO annotations) or positional gene annotations (ClinVar), raw microarray data files (GEO), microscopic images (Allen Brain Atlas), and facts pulled from published work such as affinity values for drug target interactions (UNIPROT); *Research repositories*: archives for research data files that is usually supplementary to a paper or other scholarly work such as Figshare, Dryad, or Mendeley data. *Bibliographic databases*: databases containing primarily scientific articles, research reviews, preprints, legal documents such as patents, standards, and other long text documents; for example PubMed, Scopus, Google Scholar. *Research software repository resources*: databases that are registries or repositories of compiled software, software code, coding tools, research analysis tools, software versioning systems, and code archiving tools, such as GitHub, PyPI, or Elixir’s bio. tools. *Reagent resources*: databases that serve primarily information about wet-lab and consumable research resources, such as Cellosaurus, ATCC, or the Antibody Registry.

Of the top 20 mentioned RDRs, nearly half were specialized for genomics and a quarter each for clinical research and proteomics (Fig. 2, top). RDR mentions were also clustered by type, fairly equally between research databases, bibliographic databases, research software and repository resources, reagent resources, and research repositories (Fig. 2, bottom).

### Journals with RDR mentions

We analysed RDR mentions from a journal perspective by calculating the number of all articles published in a journal mentioning at least one RDR. This approach yielded record-pairs from 3,312 journals. The distribution of journals with RDR mentions is also a long-tail type, meaning that most mentions come from a relatively small number of journals, while most journals refer only to a few RDRs. Reviewing total RDR mentions, 20 journals in the data set covered 32% of all record-pairs and the top 200 journals covered 71% of all record-pairs. This data can be skewed by journals publishing a large number of articles.

We then normalized RDR mentions by journal output and other variables, to explore which journals have the *highest proportion* of articles with any RDR mention. We adjusted for journal article volume, normalizing mentions by the number of articles published by the journal and available for mining. We selected the top 200 journals by article count as a starting subset, and ordered them by percentage of articles with at least one RDR mention, shown in Table 3. All source data can be found in Table S2.

## DISCUSSION

Our results show that mining Methods text of journal articles for RDR mentions is not only feasible, it also provides useful information that can help the community encourage and measure early-stage adoption of data sharing and reuse practices. While data sharing and reuse are not universally adopted, we show the practice is further along across the broad biological and biomedical sciences literature than DataCite or citation practices might indicate. First, using this methodology we show that authors are already engaged in using RDRs, and quantify this activity by RDR type and research area. If researchers are provided more information about how to share and reuse data—as well as more workflows to capture data mentions—we can expect more authors to mention data and RDRs in their articles. We describe a methodology for an early indicator that can be used until data citation practices are more widely adopted in the biological and biomedical community that would enable practical application of tools such as Scholix. Measuring the impact of interventions including FASEB DataWorks! community engagement combined with journal author guidance and funder policies are all necessary components in the goal of increasing research data sharing and reuse practices.

There are limitations to this approach:

- Journals vary in article volume and the availability of articles in the PubMed Central open access subset. We therefore confine our statements to describe the subset of articles in the journal that are accessible for text mining. In some cases, this subset is too small to effectively describe RDRs usage in a particular journal.
- We count only those RDRs mentioned in the Methods sections of journal articles. While many authors will refer to both their newly created and reused RDRs in that section, there may be RDR mentions in other sections of the article (including the reference list). When practical, we encourage journals to advise authors to mention their RDRs in their Methods write-up. We also encourage journals to provide guidance on use of persistent identifiers for data and RDR citations, and consider developing a journal-informed data citation policy potentially including appropriate RDRs in the reference list.
- We used the SciCrunch RRID database as a proxy for RDRs. While this is a well-curated database of over 2000 RDRs, it does not include new RDRs created during a research study. It, therefore, biases our results toward well-known and established RDRs, and may undercount actual data sharing and reuse behaviour. Extending the text mining approach to include other RDR identifiers such as accession numbers, DOIs, and other common data referents should be a next step in developing this methodology.

## CONCLUSION

Publishers, journal editors, and policymakers have several options for taking action on these findings:

- All stakeholders can be assured that data sharing and reuse is already happening and aspects of it can be tracked in the Methods section of articles.
- Publishers and journals can encourage authors to use identifiers including RRIDs to improve the unambiguous citation of RDRs and other key resources.
- Journals can use our described methodology to determine the top RDR mentions and provide targeted advice to authors in their guidelines for these specific resources.
- Journals can encourage author RDR mention behaviour through journal data sharing and reuse policies and workflows, including specific guidance on data citation (see Simons et al., 2021).
- Publishers can continue to assess RDR mentions on a regular basis as an early indicator, in combination with DataCite Event Data and/or Scholix type approaches to track adoption of data sharing and reuse behaviour. Providing article- and journal-level data citation summary results may help to promote author adoption of data sharing and reuse practices.
- Policymakers can use a variety of indicators to understand and track adoption of data sharing and reuse practices by the research community, so they can monitor the impact of their policies. We suggest they use RDR mentions as one factor in measuring compliance and in determining if policy adjustments are needed.
- Research infrastructure providers can collect and share information on data sharing and reuse using a variety of parameters, such as is happening in the Open Global Data Citation project, to support community understanding of good practice as well as promote acknowledgement for researchers who engage in data sharing and reuse activities.

## Supplementary Material

Supplemental table 2

Supplemental table 1

figure legends for supplemental tables

## Figures and Tables

**TABLE 1:** Article data set available to mine and research resources, databases, and repositories (RDR) mentions harvested.

Description	2020	2021
Total number of articles in PubMed Central for the year	1,639,036	1,756,768
Number of articles available to text mine that also have Methods (‘free to read and use’) (% off all publications)	626,395 (38%)	666,372 (38%)
Number of articles with at least one RDR mention (% of articles ‘free to read and use’)	66,187 (11%)	75,532 (11%)
Number of record-pairs (article + RDR combinations)	95,430	110,048
Number of unique RDRs mentioned	616	619

**TABLE 2:** Research resources, databases, and repositories (RDR) mentions, 2020 and 2021.

				2020		2021			
RDR name	RRID			Rank	Number of record-pairs	Rank	Number of record-pairs	Discipline/practice area	RDR type
ATCC	SCR_001672			1	15,777	1	14,514	Cell biology	Reagent repository
EMBASE	SCR_001650			2	8,772	2	10,059	Clinical research	Bibliographic database
ClinicalTrials.gov	SCR_002309			3	5,593	3	6,560	Clinical research	Bibliographic database
Addgene	SCR_002037			4	5,047	4	5,758	Genomics	Reagent repository
Cochrane Library	SCR_013000			5	4,412	6	4,984	Clinical research	Bibliographic database
Cytoscape	SCR_003032			6	4,393	5	5,265	Genomics	Research software and repository
STRING	SCR_005223			7	3,144	7	4,537	Genomics, proteomics	Research database
DAVID	SCR_001881			8	2,918	9	2,781	Genomics	Research software and repository
Ensembl	SCR_002344			9	1970	29		Genomics	Research database
REDCap	SCR_003445			10	1808	8	2,879	Clinical research	Research software and repository
PsycINFO		SCR_014799		11	1781	10	2,174	Behavioural science	Bibliographic database
Pfam		SCR_004726		12	1,598	11	1914	Proteomics	Research database
MaxQuant		SCR_014485		13	1,316	13	1,582	Proteomics	Research software and repository
Cochrane Central Register of Controlled Trials		SCR_006576	14		1,136	14	1,351	Clinical research	Bibliographic database
cBioPortal		SCR_014555	15		1,101	12	1,627	Genomics	Research repository
PANTHER		SCR_004869	16		962	17	991	Proteomics	Research database
Gene Ontology		SCR_002811	17		958	16	1,195	Genomics	Research database
miRBase		SCR_003152	18		911	20	887	Genomics	Research database
The Cancer Genome Atlas		SCR_003193	19		882	19	940	Genomics	Research database
Human Protein Atlas		SCR_006710	20		819	15	1,260	Proteomics	Reagent repository
Hmmr		SCR_005305	21			18	983	Proteomics	Research software and repository

**TABLE 3:** Journals with the greatest percentage of mineable articles having at least one research resources, databases, and repositories (RDR) mention, 2020.

Journal title	Discipline	All articles	Mineable articles	Articles with 1+ RDR mention	% mineable articles with 1+ RDR mention	Unique RDRs mentioned
*Systematic Review*	Meta-analysis, research design, systematic reviews	293	291	235	**80.8%**	16
*British Journal of Pharmacology*	Drug therapy, pharmacology	428	103	80	**77.7%**	17
*PLoS Genetics*	Genetics	567	567	354	**62.4%**	124
*EMBO Journal*	Molecular biology	314	164	95	**57.9%**	40
*Cell Reports*	Biological science disciplines	1,343	572	319	**55.8%**	96
*BMC Genomics*	Chromosome mapping, genetic techniques, genomics, sequence analysis, dna	901	898	492	**54.8%**	132
*Genome Medicine*	Genomics	110	110	55	**50.0%**	46
*Microbiome*	Environmental microbiology, microbiological phenomena	169	169	84	**49.7%**	39
*Genome Biology and Evolution*	Genomics, molecular biology	247	225	109	**48.4%**	53
*BMC Medical Genomics*	Genetics, medical genomics	196	195	92	**47.2%**	66
*Life Science Alliance*	Biological science disciplines	153	153	72	**47.1%**	33
*mSystems*	Microbiological phenomena	328	328	151	**46.0%**	67
*Cell*	Biochemical phenomena, biophysical phenomena, cells	608	165	73	**44.2%**	55
*Genome Biology*	Genomics, molecular biology	304	304	134	**44.1%**	90
*BMC Microbiology*	Microbiological techniques, microbiology	372	370	163	**44.1%**	50
*Journal of Experimental & Clinical Cancer Research*	Medical oncology	287	285	125	**43.9%**	37
*Oncogene*	Oncogenes	457	213	92	**43.2%**	34
*Microbial Genomics*	Genome, microbial genomics	156	156	67	**42.9%**	50
*G3 (Bethesda)*	Genes, genetics, genomics	421	409	171	**41.8%**	92
*Cancer Cell International*	Cell transformation, neoplastic neoplasms	605	598	250	**41.8%**	50

## Data Availability

The data that support these findings of the study are provided as Supplementary tables referenced in the Methods section text and openly available in figshare at: Table S1—https://doi.org/10.6084/m9.figshare.22720399 and Table S2—https://doi.org/10.6084/m9.figshare.22720399.

## Key points

- We developed and tested a model for measuring data sharing and reuse that uses text mining of methods sections for mentions of RDRs in biological and biomedical research articles.
- We found that authors publishing in biological and biomedical sciences are mentioning RDRs in the description of their Methods.
- We provide baseline statistics for author mentions of RDRs for 2020 and 2021, normalized by journal volume, and with detail on discipline and RDR type.
- We propose this approach be used as one early indicator for tracking data sharing and reuse patterns at the journal and discipline level over time.

## Footnotes

^1^We worked with DataCite in August 2022 to understand data citation behaviour using the DataCite Event Data API. There are six relation Types that can count as citations for a given DOI A (see https://support.datacite.org/docs/contributing-citations-and-references): DOI A IsCitedBy, IsSupplementTo, or IsReferencedBy another DOI B; or DOI B Cites, Reference, or IsSupplementedBy DOI A. Of the over 32 million DataCite DOIs, 370,674 had at least one citation (https://api.datacite.org/dois/?query=-citationCount:0). In 2020, there were 5,854 DOIs registered with DataCite of type ‘data set’ with at least one citation (https://api.datacite.org/dois?resource-type-id=Dataset&published=2020&query=-citationCount:0) the majority of which were registered post-publication by article repositories. The entire 2020 DataCite Event set had over a million citations, over 95% of which were associated with a single repository.

^2^https://europepmc.org/search?query=%28FIRST_PDATE%3A%5B2020–01-01%20TO%202020–12-31%5D%29&page=1, data collected in January 2023. Note that the category ‘free to read’ is free to read by a human and is not always sufficient for text mining.
