# Supplementary material for: Establishing an early indicator for data sharing and reuse
Source: Learn Publ. Author manuscript; Available in PMC 2025 Aug 12. (PMC12341665; doi:10.1002/leap.1586)
Supplement: figure legends for supplemental tables [file NIHMS2043515-supplement-figure_legends_for_supplemental_tables.rtf]

leap1586-sup-0001-Supplementary_Table_1.xlsxExcel 2007 spreadsheet , 1.4 MB	Supplementary Table S1. is a discrete subset of SciCrunch RDRs used to study RDR mentions in biomedical literature. We generated this list by starting with the top 1,000 entries in the SciCrunch database, measured by citations, removed entries for organizations (such as universities without a corresponding RDR) or non-relevant tools (such as reference managers), updated links, and consolidated duplicates resulting from RDR mergers and name variations. The resulting list of 737 RDRs is shown in with as a base based on a source list of RDRs in the SciCrunch database. he file includes the Research Resource Identifier (RRID), the RDR name, and a link to the RDR record in the SciCrunch database.	
leap1586-sup-0002-Supplementary_Table_2.xlsxExcel 2007 spreadsheet , 13.1 MB	Supplementary Table S2. hows the RDRs, associated journals, and article-mention pairs (records) with text snippets extracted from mined Methods text in 2020 PubMed articles. The data set has 4 components. The first shows the list of repositories with RDR mentions, and includes the Research Resource Identifier (RRID), the RDR name, the number of articles that mention the RDR, and a link to the record in the SciCrunch database. The second shows the list of journals in the study set with at least 1 RDR mention, and includes the Journal ID, name, ESSN/ISSN, the total count of publications in 2020, the number of articles that had text available to mine, the number of article-mention pairs (records), number of articles with RDR mentions, the number of unique RDRs mentioned, % of articles with minable text. The third shows the top 200 journals by RDR mention, normalized by the proportion of articles with available text to mine, with the same metadata as the second table. The fourth shows text snippets for each RDR mention, and includes the RRID, RDR name, PubMedID (PMID), DOI, article publication date, journal name, journal ID, ESSN/ISSN, article title, and snippet.	
